# Hypercitratemia is a mortality predictor among patients on continuous venovenous hemodiafiltration and regional citrate anticoagulation

**DOI:** 10.1038/s41598-023-47644-1

**Published:** 2023-11-17

**Authors:** Thais Oliveira Claizoni dos Santos, Carlos Eduardo dos Santos Ferreira, Cristóvão Luis Pitangueira Mangueira, Adriano Luiz Ammirati, Patricia Faria Scherer, Marisa Petrucelli Doher, Thais Nemoto Matsui, Bento Fortunato Cardoso dos Santos, Virgílio Gonçalves Pereira, Marcelo Costa Batista, Julio Cesar Martins Monte, Oscar Fernando Pavão Santos, Marcelino de Souza Durão

**Affiliations:** 1 Nephrology Division, Universidade Federal de São Paulo, Rua Botucatu, 740, São Paulo, SP 04023-062 Brazil; 2 School of Medicine, Universidade de Pernambuco, Recife, PE Brazil; 3 Clinical Laboratory, Hospital Israelita Albert Einstein, São Paulo, SP Brazil; 4 Nephrology Division, Hospital Israelita Albert Einstein, São Paulo, SP Brazil; 5 Dialysis Center, Hospital Israelita Albert Einstein, São Paulo, SP Brazil; 6 Intensive Care Unit, Hospital Israelita Albert Einstein, São Paulo, SP Brazil; 7 School of Medicine, Faculdade Israelita de Ciências da Saúde Albert Einstein, São Paulo, SP Brazil; 8 Kidney Transplant Unit, Hospital Israelita Albert Einstein, São Paulo, SP Brazil

**Keywords:** Nephrology, Renal replacement therapy

## Abstract

The use of regional citrate anticoagulation (RCA) in liver failure (LF) patients can lead to citrate accumulation. We aimed to evaluate serum levels of citrate and correlate them with liver function markers and with the Cat/Cai in patients under intensive care and undergoing continuous venovenous hemodiafiltration with regional citrate anticoagulation (CVVHDF-RCA). A prospective cohort study in an intensive care unit was conducted. We compared survival, clinical, laboratorial and dialysis data between patients with and without LF. Citrate was measured daily. We evaluated 200 patients, 62 (31%) with LF. Citrate was significantly higher in the LF group. Dialysis dose, filter lifespan, systemic ionized calcium and Cat/Cai were similar between groups. There were weak to moderate positive correlations between Citrate and indicators of liver function and Cat/Cai. The LF group had higher mortality (70.5% vs. 51.8%, *p* = 0.014). Citrate was an independent risk factor for death, OR 11.3 (95% CI 2.74–46.8). In conclusion, hypercitratemia was an independent risk factor for death in individuals undergoing CVVHDF-ARC. The increase in citrate was limited in the LF group, without clinical significance. The correlation between citrate and liver function indicators was weak to moderate.

## Introduction

Critically ill patients often require dialysis. In this context, patients with multiple organ dysfunction, including liver failure (LF), are recommended continuous kidney replacement therapy (CKRT)^1^. Regional citrate anticoagulation (RCA) is the anticoagulation method of choice during CKRT and when compared to heparin, RCA prolongs the filter lifespan, reduces the bleeding risk, and decreases the need for transfusion^2–9^.

But a limitation to the use of citrate is when mitochondrial metabolism is reduced, especially in the liver and muscles. This occurs especially in LF and in decreased muscle perfusion, such as in cases of severe and prolonged shock^7^. Citrate accumulation is characterized by high anion gap metabolic acidosis due to elevated blood citric acid level^10,11^, systemic ionized hypocalcemia, increased systemic total calcium to ionized calcium ratio (Cat/Cai) and systemic levels and elevated serum citrate^12–15^. Some studies suggest that a Cat/Cai ratio greater than 2.5 is indicative of citrate accumulation^12,16,17^. However, this cut-off value was not able to discriminate all individuals who had high blood citrate levels^12,14,15,17^.

Therefore, this study aimed to evaluate the serum levels of citrate and correlate them with liver function markers and with the Cat/Cai ratio in patients undergoing continuous venovenous hemodiafiltration with regional citrate anticoagulation (CVVHDF-RCA). We compared patients with and without LF, assessing the mortality rate in 90 days and other associated risk factors.

## Methods

### Study design and setting

We conducted a prospective cohort study at Hospital Israelita Albert Einstein, in São Paulo, Brazil, with ICU patients recruited from January 2013 until April 2014.

### Participants

We included in this study all consecutive patients aged 18 years or older admitted to the ICU between January 2013 and April 2014, with acute or chronic kidney failure, submitted to CVVHDF-RCA by recommendation of the nephrology team. We excluded patients that did not complete 24 h of therapy or refused to participate in the study. We considered patients as in hepatic failure when the international normalized ratio (INR) was equal to or greater than 2.5 for a period of at least 48 h, due to known liver disease or multiple organ failure^18^.

### Exposures

We measured the blood citrate level before starting and during the first 7 days of CVVHDF.

### Statement of ethics

This study followed the Helsinki Declaration principles and the study protocol was evaluated and approved by the Hospital’s Institutional Review Board (protocol number 162712). All patients or their guardians signed informed consent forms for the use of the data in this study.

### Variables

The relevant data for our study were extracted in an anonymized way from medical record review, using a prespecified data collection form. We evaluated baseline clinical and personal characteristics of the patients such as age, sex, ethnicity, reason for admission to the ICU and comorbidities. We also collected data during the ICU admission, such as the need for mechanical ventilation, use of vasopressors, etiology of renal dysfunction. We calculated the Simplified Acute Physiology Score (SAPS) 3, at the time of ICU admission, and Sequential Organ Failure Assessment (SOFA) scores in the first 7 days of dialysis (including renal SOFA values).

Systemic ionized calcium and post-filter ionized calcium from the dialysis circuit, were evaluated every 6 h. Sodium, potassium, arterial blood gases were collected every 12 h. Blood count, prothrombin time, transaminases and bilirubin, activated partial thromboplastin time, phosphorus, magnesium, total calcium, creatinine, systemic urea, pre-filter urea and effluent urea were collected every 24 h in the morning. Serum citrate was measure daily using the citrate-lyase method (Citric acid UV, R-Biopharm AG, Darmstadt, Germany), as described in previous studies^17,19^.

We registered the duration of dialysis treatment (in hours), as well as blood flow, dialysate, replacement solution, citrate, and calcium infusion. The filtration fraction and the dialysis dose were calculated daily, as well as the ratio between effluent urea/pre-filter urea (to check the filter’s patency) and filter life (time in hours of use), and we registered these data. We also evaluated filter change due to clotting.

### Outcome variables

We assessed 90-day mortality after initiation of CVVHDF and factors related to death as the primary outcomes. The secondary outcomes evaluated were: length of stay in the ICU and hospital, time in CVVHDF, occurrence of electrolyte and acid–base balance disorders during the period on dialysis, need for transfusion, useful lifespan of filters and the number of filter changes due to coagulation.

### Data sources/measurements

As a routine in the service, systemic Ca and post-filter ionized Ca, are evaluated every 6 h, and we registered these data. Sodium, potassium, arterial blood gases were collected every 12 h. Blood count, prothrombin time, transaminases and bilirubin, activated partial thromboplastin time, phosphorus, magnesium, total calcium, creatinine, systemic urea, pre-filter urea and effluent urea were collected every 24 h in the morning. For this study, the measurement of serum citrate level was added to the daily morning routine. Serum citrate was measured using the citrate-lyase method (Citric acid UV, R-Biopharm AG, Darmstadt, Germany), as described in previous studies^17,19^.

### Continuous venovenous hemodiafiltration treatment protocol

The regional citrate anticoagulation for continuous venovenous hemodiafiltration, (CVVHDF) in acute kidney injury protocol used in this work was described in a previous publication^18^. CVVHDF was performed using a Prismaflex machine (Baxter), with an M100 hemofilter and an AN69 membrane (Baxter).

An ultrasound-guided vascular access was achieved by the insertion of an 11.5 F three-lumen venous catheter (Arrow International, PA, United States of America) into the internal jugular vein, preferably on the right, or femoral veins. Blood flow was kept constant at 100 ml/min. A 4% trisodium citrate solution and a calcium replacement solution (0.75% CaCl_2_) were infused into the arterial line (via a three-way stopcock) and into the third port of the dialysis catheter, respectively.

The initial citrate and calcium solutions infusion flow was 150 ml/h and 80 ml/h, respectively. The citrate solution infusion was adjusted according to the electronic spreadsheet, aiming to maintain the post-filter ionized calcium concentration in the range between 0.25 and 0.30 mmol/l. Likewise, the calcium solution infusion was adjusted in order to maintain the systemic concentration of ionized calcium between 1.12 and 1.20 mmol/l. However, in patients with LF (INR ≥ 2.5), the initial citrate flow was 120 ml/h and kept fixed at this rate regardless of the post-filter ionized calcium value.

Sodium bicarbonate (8.4%) and potassium phosphate (2 mEq/ml) were added as needed to the dialysis solution, that contained sodium 110 mEq/l, chloride 111.5 mEq/l, magnesium 1.5 mEq/l and dextrose 0.1%. Regarding the replacement solution (0.45% NaCl), 20% NaCl and 10% MgSO_4_ were added as needed. These electrolytes were added to the bags by the hospital pharmacy.

The prescribed dialysis dose was 35 ml/kg/h (total volume of effluent) on average, to ensure a minimum effective dose of 25 ml/kg/h, thus compensating for a possible decrease in efficiency over time (fiber coagulation) and premature interruptions of therapy for tests or other procedures. The prescription made sure that two thirds of the dose was diffusive clearance and one third was convective clearance.

The filter and extracorporeal circuit were replaced every 72 h, or earlier in case of clotting or for exams or surgeries.

### Statistical analysis

We describe qualitative variables by absolute frequencies and percentages. We give quantitative variables by means and their standard deviations or medians and interquartile ranges, where appropriate, after verifying the distribution by histograms and Shapiro–Wilk normality tests, using a complete case analysis. We verified associations between categorical variables using the Fisher exact test and compared groups for numerical variables using Mann–Whitney’s when distribution was not normal. We compared them using the Spearman correlation coefficient. We estimated survival up to 90 days using the Kaplan–Meier method.

We used logistic regression models, with simple and multiple approaches, to analyze the factors associated with death. Variables with a *p*-value lower than 0.2 in the bivariate analysis were eligible for the multivariable model. After multivariable adjustment, only variables with a p-value lower than 0.2 were kept. We used logistic regression in the model and predicted accuracy using C-statistics (area under the curve). For the comparison of survival between patients with and without liver failure, with used the likelihood test. We present the results using odds rations with 95% confidence intervals and p-values. We also analyzed the behavior of continuous variables applying a linear model to longitudinal data GEE (generalized estimating equation model).

We used the SPSS software, version 18 (SPSS Inc, Chicago, IL) and Stata, version 14 (StataCorp. 2015. Stata Statistical Software: Release 14. College Station, TX: StataCorp LP) in the analyzes. We considered as significant the *p*-values of 0.05 and below.

## Results

### Participants flow

Between January 2013 and April 2014, 212 adult patients were admitted to the ICU with acute or chronic kidney failure and submitted to CVVHDF-RCA. We excluded 5 patients who did not complete 24 h of therapy and another 7 who refused to participate in the study. The demographic and clinical characteristics of the 200 included in the study are described in Table 1, that shows 17 patients (8.5%) as chronic kidney patients in regular dialysis programs, and sepsis as the main reason for ICU admission.

**Table 1:** Baseline demographic and clinical characteristics.

	Liver failure			*p*-value
	Yes (62)	No (138)	Total (200)	
Male sex	35 (56.5)	90 (65.2)	125 (62.5)	0.236
Age (median, years)	60.5 (51.8–66.3)	70 (57.8–82)	66 (55–78)	< 0.001
White ethnicity	52 (83.9)	126 (91.3)	178 (89)	0.120
SAPS-3 (at ICU admission)	60 (48.5–72)	63 (55.75–77)	62 (51–75)	0.10
SOFA				
First CVVHDF day	15 (13–17)	12.5 (19.75–14)	13 (11–15)	< 0.001
Second CVVHDF day	16 (13–17)	12 (10–14)	13 (11–15)	< 0.001
Third CVVHDF day	16 (13–17)	12 (10–14)	13 (11–15)	< 0.001
Fourth CVVHDF day	15 (13–17)	12 (9–14)	13(10–15)	< 0.001
Fifth CVVHDF day	16 (14–18)	11 (9–14)	13 (10–16)	< 0.001
Sixth CVVHDF day	15 (14–17)	11(8–14)	13(10–15)	< 0.001
Seventh CVVHDF day	16 (14–17)	11 (9–14)	13(10–16)	< 0.001
Comorbidities				
CKD on dialysis	5 (8.1)	12 (8.7)	17 (8.5)	0.882
Systemic arterial hypertension	13 (21)	54 (39.1)	67 (33.5)	0.015
Diabetes mellitus	13 (21)	38 (27.5)	51 (25.5)	0.382
Chronic obstructive pulmonary disease	4 (6.5)	20 (14.5)	24 (12)	0.157
Solid malignant tumor	7 (11.3)	22 (15.9)	29 (14.5)	0.516
Hematological cancer	4 (6.5)	22 (15.9)	26 (13)	0.072
HSCT	1 (1.6)	9 (6.5)	10 (5)	0.178
Cirrhosis	28 (45.2)	14 (10.1)	42 (21)	< 0.001
Solid organ transplant				
Liver	16 (25.8)	19 (13.8)	35 (17.5)	0.038
Heart	1 (1.6)	2 (1.4)	3 (1.5)	0.930
Kidney	1 (1.6)	6 (4.3)	7 (3.5)	0.440
Reason for admission to the ICU				
Sepsis	31 (50)	82 (59.4)	113 (56.5)	0.214
Liver failure	9 (14.5)	4 (2.9)	13 (6.5)	0.002
Heart failure	2 (3.2)	10 (7.2)	12 (6)	0.349
Solid organ transplant	10 (16.1)	11 (8)	21 (10.5)	0.082
Elective surgery	3 (4.8)	10 (7.2)	13 (6.5)	0.523
Emergency surgery	4 (6.5)	16 (11.6)	20 (10)	0.262
Vasoactive drug use	54 (87.1)	115 (83.3)	169 (84.5)	0.496
Mechanical ventilation	53 (85.5)	105 (76.1)	158 (79)	0.131
Sedation	50 (80.6)	98 (71)	148 (74)	0.151
Causes of liver failure				
Multiple organ dysfunction	17 (27.4)	–	–	–
Cirrhosis	32 (51.6)	–	–	–
Related to liver transplant	13 (20.9)	–	–	–

ICU, intensive care unit; SAPS, Simplified Acute Physiology Score; SOFA, Sequential Organ Failure Assessment; CCVVHDF, continuous venovenous hemodiafiltration; CKD, chronic kidney disease; HSCT, hematopoietic stem cell transplantation.

From the 200 patients, 62 (31%) had LF, with 32 (51.6%) with cirrhosis. In another 17 cases (27.4%), liver failure occurred as a component of multiple organ and system dysfunction and in 13 (20.9%) as a result of complications in liver transplant recipients.

### Lab results

Table 2 shows the lab results immediately before starting CVVHDF (medians and interquartile ranges, IQR), comparing patients with and without LV. Patients with liver failure had significantly lower pH, serum bicarbonate levels, systemic ionic calcium, hemoglobin and platelets. They also had higher values of arterial lactate, aspartate aminotransferase (AST), alanine aminotransferase (ALT), total bilirubin, international normalized ratio (INR) and activated partial thromboplastin time (APPT). Lab data from the third and seventh day of CVVHDF are shown in Supplementary Table, S1 and S2 respectively.

**Table 2:** Lab results before starting continuous venovenous hemodiafiltration (medians and interquartile ranges, IQR).

	Liver failure			*p*-value
	Yes (62)	No (138)	Total (200)	
Creatinine (mg/dl)	2.7 (1.8–3.5)	2.7 (1.9–3.7)	2.7 (1.91–3.71)	0.579
Urea (mg/dl)	99 (61–148)	114 (84–153)	112 (78–151)	0.119
Basal sodium (mEq/l)	139 (134–142)	139 (135–143)	139 (135–143)	0.968
Basal potassium (mEq/l)	4.4 (4.0–5.0)	4.3 (3.8–5.0)	4.3 (3.9–5.0)	0.3125
Chloride (mEq/l)	105 (100–108)	104 (99–110)	104 (99–108)	0.736
Anion gap	18 (14–26)	16 (12–19)	16 (13–21)	0.196
Systemic ionized calcium (mmol/l)	1.06 (1.02–1.14)	1.10 (1.06–1.16)	1.10 (1.04–1.15)	0.019
Total calcium (mg/dl)	8.4 (7.7–8.9)	8.2 (7.8–8.7)	8.2 (7.9–8.8)	0.920
Phosphor (mg/dl)	5.7 (4.3–6.7)	4.7 (3.7–6.5)	4.7 (3.8–6.6)	0.252
Magnesium (mg/dl)	1.6 (1.4–2.0)	1.6 (1.4–1.8)	1.6 (1.4–1.88)	0.615
pH	7.32 (7.26–7.38)	7.37 (7.29–7.43)	7.35 (7.27–7.41)	0.011
Bicarbonate (mEq/l)	15.9 (12.7–20.5)	19.4 (16.8–23.2)	18.6 (15.2–22.1)	< 0.001
Lactate (mg/dl)	33.5 (17.8–83)	16.0 (12–26)	19 (13–35)	< 0.001
AST (U/l)	203 (51–3180)	70 (30–241)	79 (36–482)	0.03
ALT (U/l)	282 (42–2658)	49 (31–115)	62 (34–308)	0.01
Total bilirubin (mg/dl)	7.7 (4.7–11.2)	1.3 (0.6–4.7)	2.9 (0.9–7.8)	< 0.001
Direct bilirubin (mg/dL)	4.8 (2.9–8.2)	0.9 (0.4–3.0)	2.1 (0.7–5.4)	< 0.001
INR	2.68 (2.24–3.63)	1.42 (1.27–1.73)	1.7 (1.33–2.37)	< 0.001
APPT ratio	1.72 (1.54–2.1)	1.28 (1.13–1.59)	1.44 (1.19–1.75)	< 0.001
Hemoglobin (g/dl)	8.1 (7.1–9.3)	9.1 (8.1–10.5)	8.9 (7.8–10.1)	< 0.001
Hematocrit (%)	23.1 (21.1–28.2)	27.1 (23.9–30.7)	26.3 (22.1–30)	< 0.001
Platelets × 10^3^ (n/µl)	51.5 (35.8–51.5)	109 (48–179)	77 (42–155)	< 0.001

AST, aspartate aminotransferase; ALT, alanine aminotransferase; INR, international normalized ratio; APPT, activated partial thromboplastin time.

Table 3 shows the median levels of citrate up to the seventh day of dialysis using CVVHDF, comparing the patients with and without LF. Although baseline values were similar between groups, from the first day of dialysis on there were significant differences comparing patients with and without LV.

**Table 3:** Median citrate levels (in mg/dL) in patients with and without liver failure (and interquartile ranges, IQR).

Citrate (mg/dl)*	Liver failure						*p*-value
	Yes (57)	N	No (128)	N	Total (185**)	N	
Basal	2.95 (2.12–3.78)	57	2.43 (1.81–3.54)	128	2.58 (1.90–3.59)	185	0.022
First day of dialysis	3.57 (2.83–4.82)	55	2.87 (2.35–4.50)	121	3.12 (2.43–4.53)	176	0.006
Second day of dialysis	3.56 (2.73–4.82)	52	2.95 (2.31–4.30)	113	3.07 (2.39–4.40)	165	0.007
Third day of dialysis	3.59 (2.76–4.87)	46	2.81 (2.24–3.82)	98	2.99 (2.32–4.06)	144	0.001
Fourth day of dialysis	3.83 (2.83–4.62)	38	2.83 (2.12–3.72)	80	2.99 (2.24–4.00)	118	0.005
Fifth day of dialysis	3.68 (2.49–4.74)	34	2.63 (1.95–3.61)	67	2.83 (2.16–4.01)	101	0.005
Sixth day of dialysis	3.35 (2.67–4.56)	32	2.82 (2.05–4.03)	57	3.09 (2.23–4.17)	89	0.037
Seventh day of dialysis	3.95 (2.68–5.39)	28	2.68 (2.26–3.99)	43	3.04 (2.33–4.40)	71	0.020

*To convert citrate from mg/dl to µmol/l, multiply it by 52.08 (normal values: 0.96–2.50 mg/dl or 50–130 µmol/l). **Patients with missing values were excluded (complete case analysis).

**Figure 1:**
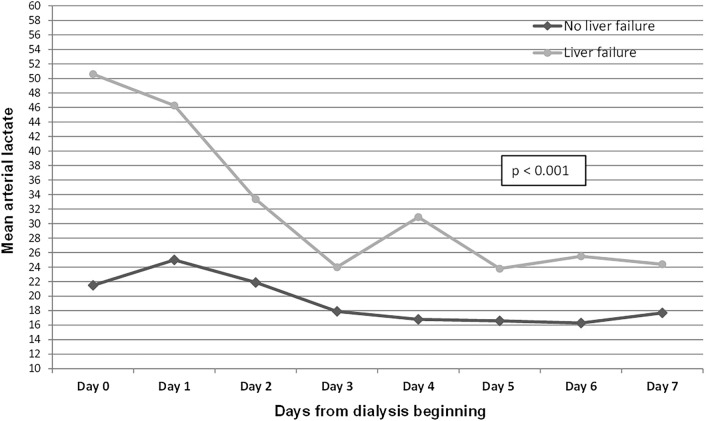
Generalized estimating equation (GEE) model of the variation of arterial lactate according to liver failure in the seven first days of dialysis.

**Figure 2:**
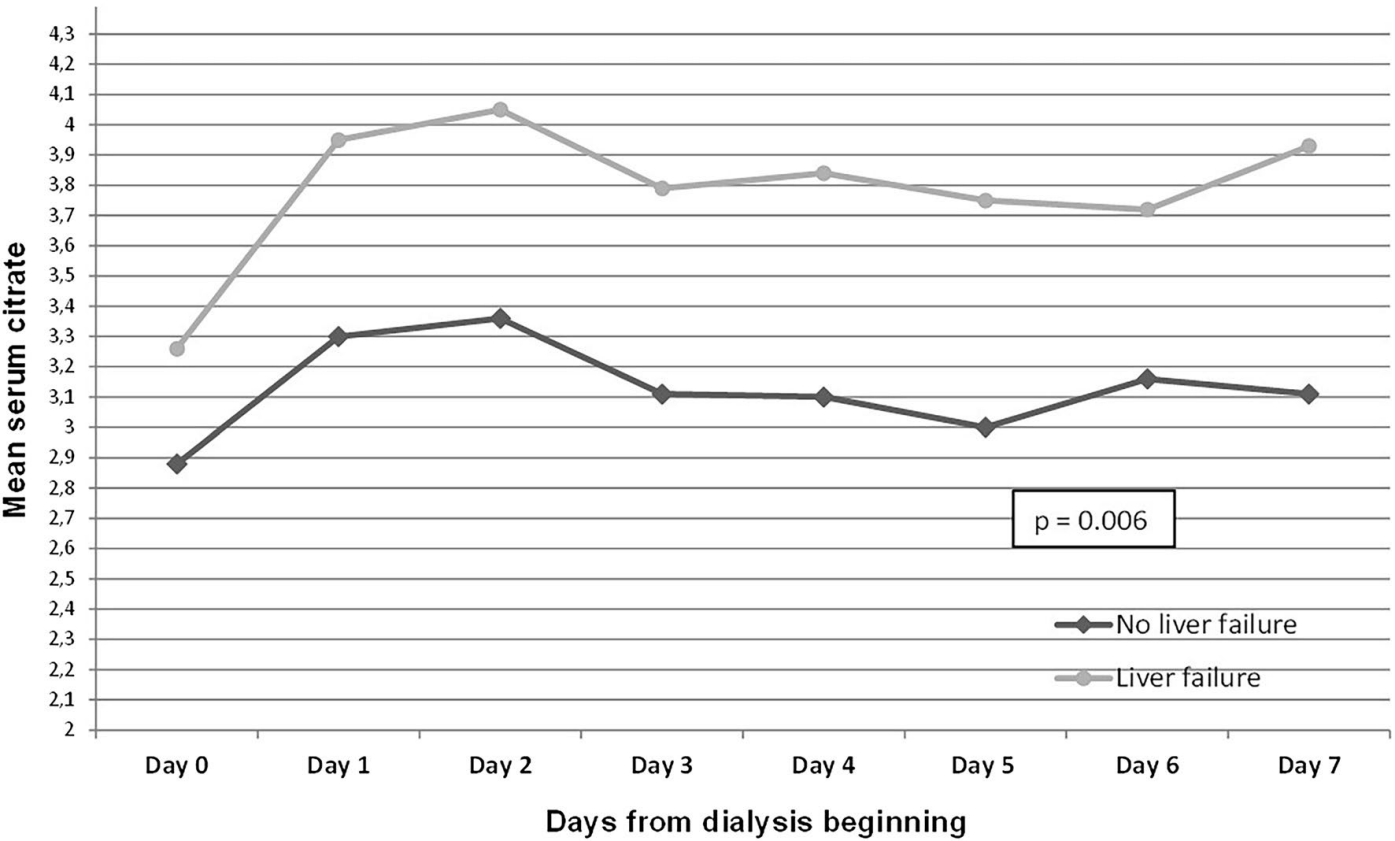
Generalized estimating equation (GEE) model of the variation of serum citrate according to liver failure in the seven first days of dialysis.

According to the GEE model, mean levels of levels of lactate and citrate were significantly higher along the first 7 days of dialysis in patients with LF. These results are presented in Figs. 1, 2. Mean levels of bicarbonate and pH were significantly lower and serum sodium did not present significant changes during the 7 days of dialysis (see Supplementary Fig. 1, 2 and 3, respectively).

We evaluated the frequencies of electrolyte and acid–base serum changes in patients with and without LV (shown in Supplementary Table S3). This analysis totalized 1267 observations, with 417 observations among patients with LF. Abnormally elevated pH (7.50 or higher), elevated potassium (6.0 mEq/l or higher), and elevated bicarbonate (30 mEq/l or higher) were significantly less frequent in patients with LF; while reduced bicarbonate (15 mEq/l or lower) were more frequent in LF.

Except for the observation on the first day, the median values of total and ionized calcium were similar between patients with and without LF along the first 7 days of dialysis. These results are shown in Supplementary Table S4.

The correlation between serum citrate measurements and total calcium and ionized calcium ratio, international normalized ratio (INR), total bilirubin, arterial lactate, and aspartate aminotransferase (AST) is shown in Supplementary Tables S5 to S9. When significant, the correlations were weak or moderate, with the highest correlations observed with total bilirubin.

### Dialysis data analysis

As shown in Table 4, the prescribed and convective dose of dialysis was similar between patients with and without LF. Citrate flow was significantly lower among patients with LF. Data about time under dialysis and the lifespan of filters was also similar between groups.

**Table 4:** Dialysis and filter output values for patients with and without liver failure*

	Liver failure			*P*-value
	Yes (62)	No (138)	Total (200)	
Blood flow (ml/min)	100 (100–100)	100 (100–100)	100 (100–100)	0.94
Effluent flow (ml/h)	2918 (2837–3029)	2906 (2792–3068)	2912 (2809–3058)	0.691
Calcium flow (mmol/h)	4.47 (4.13–4.8)	4.53 (4.2–4.75)	4.5 (4.20–4.76)	0.423
Citrate flow (mmol/h)	17.3 (16.3–20.5)	24.4 (23.4–25.5)	23.6 (20.3–25.0)	< 0.001
Prescribed dose (ml/kg/h)	36.8 (32.2–44.3)	39 (33.9–44)	38 (33.5–44)	0.315
Convective dose (ml/kg/h)	11.1 (9.7–13.5)	12 (10–14)	11.7 (9.9–13.7)	0.205
Delivered dose (ml/kg/h)	32 (28.3–38.9)	34.9 (29.9–39.6)	33.4 (28.9–39.6)	0.141
Filtration fraction	0.19 (0.17–0.20)	0.19 (0.18–0.21)	0.19 (0.18–0.21)	0.20
Pre-filter urea/effluent urea	0.88 (0.85–0.)	0.89 (0.87–0.91)	0.89 (0.86–0.91)	0.043
Number of filters per patient	3 (1–4.25)	2 (1–4)	2 (1–4)	0.229
Filter changes due to coagulation^a^	48 (12.2)	27 (13.9)	75 (12.8)	0.560
Filter lifespan (h)	60.5 (44.8–69.7)	62.3 (43.2–71.7)	61 (44.3–71.1)	0.832
Interruption time (h)	3 (0–15.4)	1.5 (0–9.1)	2 (0–12)	0.176
Total time of CVVHDF (h)	145.5 (80–244.3)	122.5 (70.2–222.8)	132.3 (71.3–229.9)	0.21

*Flows and doses shown as means during dialysis; other variables shown as medians and interquartile ranges (Q1-Q3).

^a^Total number of filters used: 587 (194 in patients with and 393 in patients without liver failure).

CVVHDF, continuous venovenous hemodiafiltration.

### Clinical outcomes and survival

**Figure 3:**
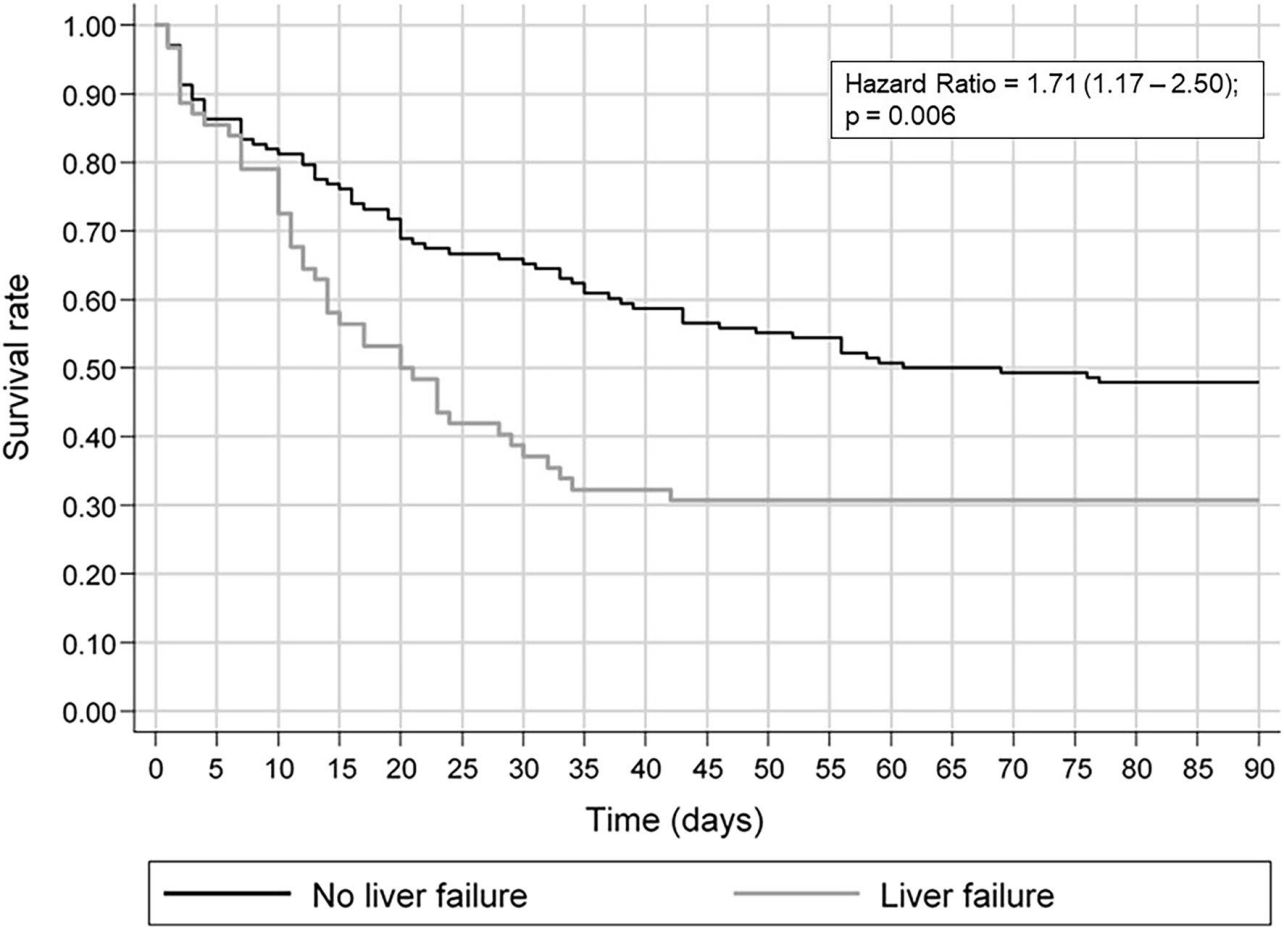
Survival curve in 90 days in patients with and without liver failure.

Overall, 115 patients (57.5%) died; 43 (69.4%) with and 72 (52.2%) without LF, respectively (*p* = 0.023). The time on CVVHDF was similar between the groups with and without LF, of 7 (3.75–10.25) and 5.5 (3–10) days, respectively (*p* = 0.192). Figure 3 shows the survival curve up to 90 days after the start of dialysis, showing that LF had a significant impact on survival (*p* = 0.004). There were 18.4 deaths per 1,000 dialysis patients per day (95% CI 13.6–24.8) among patients with liver failure and 9.5 (95% CI 7.5–11.9) among patients without LF.

By estimating the hazard ratio, the risk of death of a patient with LF increased by 1.71 times within 90 days after the beginning of CVVHDF (*p* = 0.006).

Supplementary Table S10 shows demographic and clinical characteristics of patients who died versus those who survived. It shows that higher SAPS-3 and SOFA scores, hematological cancer, and cirrhosis as significantly associated with death, while a liver transplant was associated with survival. Most the reasons for the admission to the ICU were also linked to death. No patient without LF needed platelet transfusions, and only 1 patient in liver failure required it during CVVHDF (*p* = 0.074). However, the need for red blood cells transfusions was significantly different between groups [3 (95% CI 0.5–5) and 1 (0–2); *p* < 0.001, in patients with and without LF respectively]. Two patients with LF needed fresh or cryoprecipitate plasma transfusions (95% CI 0–4.5) and none in the group without LF (*p* < 0.001).

Table 5 presents the multivariate analysis, and it identifies serum citrate level change as an independent risk factor for death. The risk of dying with abnormal citrate levels was around 11 times higher for each increase in citrate unit.

**Table 5:** Association between clinical and laboratorial factors and death.

Explanatory variables	Univariate analysis OR (IC 95%)	*P*-value	Multivariate analysis OR (IC 95%)	*p*-value
COPD	2.44 (0.93–6.45)	0.071	2.97 (0.95–9.27)	0.061
Cirrhosis	2.48 (1.16–5.28)	0.018	4.49 (1.85–10.9)	0.001
Hematological cancer	2.77 (1.06–7.23)	0.037	4.13 (1.23–13.9)	0.022
SAPS-3^a^	1.03 (1.01–1.05)	< 0.001	1.02 (1.00–1.04)	0.030
Vasoactive drug use	2.11 (0.97–4.58)	0.060	3.09 (1.06–9.04)	0.039
Mechanical ventilation	2.73 (1.35–5.49)	0.005	2.46 (1.03–5.89)	0.043
Citrate change*^a^	7.21 (2.38–21.8)	< 0.001	11.3 (2.74–46.8^a^	0.001

COPD, chronic obstructive pulmonary disease; SAPS, simplified acute physiology score.

*Mean change in serum citrate level throughout dialysis.

^a^Relationship between the chance of death and an increase of one unite of the variable.

## Discussion

### Citrate and liver function

Liver failure is the main obstacle to the use of regional citrate anticoagulation (RCA) in critically ill patients, as there is a risk of citrate accumulation generating electrolyte and acid–base balance disturbances. However, we showed, in our study, that hypercitratemia was a risk factor for death regardless of cirrhosis, the main cause of LF. Although patients with LF had higher levels of citrate than those without liver dysfunction, we did not observe laboratory signs of significant accumulation. Furthermore, blood levels of citrate were weakly correlated with the main LF biomarkers used in clinical practice. Even before the start of CVVHDF and the RCA protocol, individuals with LF already had higher blood levels of citrate than those without LF, and this phenomenon persisted during the first week of dialysis, showing a possible metabolism deficiency.

The mean values of serum citrate levels in our study were lower than those found by others. In a small study with 16 patients in Germany, Hetzel et al.^17^ patients undergoing CVVHDF had serum citrate levels of 16 mg/dl on the third day of dialysis. In another German study, which included 43 patients with LF who underwent CVVHDF and a citrate flow of 4 mmol/l of blood, a peak in the serum citrate level was observed after 72 h of therapy, with a median of 16 mg/dl, reaching values up to 29 times higher than baseline^16^. In our study, the mean blood citrate level during the first 7 days of CVVHDF-RAC was 3.65 and 3.01 mg/dl in the groups with and without LF, respectively. Perhaps, differences in the criteria for defining LF and disease severity, the clearance method used, and the dialysis dose could explain the discrepancies between our findings and the others.

Citrate is a water-soluble substance, very dialysable and, like urea, has a sieving coefficient around 1.0. More than half of the infused citrate load is cleared by the dialysis system during RCA^19,20^. The remainder needs to be metabolized within the Krebs cycle, mainly in the liver. We did not observe significant accumulation of citrate probably because we set the citrate infusion at 2.5 mmol/l of blood in the group with LF and reached the real dialysis dose around 30 ml/kg/h, where two thirds of this was as diffusive clearance. The Cat/Cai ratio was not different between patients with and without LF (with the exception of the first day) and we did not observe a difference in Ca replacement or systemic ionized Ca levels during the first week of CVVHDF and RCA.

### Citrate and dialysis data

As we do not routinely measure citrate levels, we use the Cat/Cai ratio as an indicator of citrate accumulation. It is speculated^12,16,17^ that values greater than 2.5 would identify individuals with accumulation and risk of developing complications such as systemic ionic hypocalcemia and metabolic acidosis. Kramer et al. evaluated the pharmacokinetics of intravenous infusion of trisodium citrate in subjects with and without cirrhosis and showed that the Cat/Cai ratio significantly correlated with serum citrate levels in both groups. This variable, however, identified only 3 of the 15 samples from cirrhotic patients with citrate values greater than 28.8 mg/dl^14^. In our study, we found a positive but weak relationship between blood citrate levels and the Cat/Cai ratio (mean during the first 7 days of CVVHDF r = 0.280). More recently, Klingele et al. suggested that the Cat/Cai ratio could be an indicator of disease severity and mortality^21^.

We also found a weak to moderate the correlation between citrate levels and the main indicators of liver function used in clinical practice, such as INR and bilirubin. The variable total bilirubin showed the highest correlation (mean during the first 7 days of CVVHDF r = 0.509). For INR, this was 0.401. However, citrate dosing is not a routine in all ICUs.

We observed that individuals with LF had acidosis and higher levels of lactate when compared to those without LF. Hyperlactatemia can result from increased production, mitochondrial dysfunction, an imbalance between oxygen supply and demand, and a primary decrease in its use, as it happens in liver disease^22,23^. Tan et al.^24^ showed that patients with signs of citrate accumulation (Cat/Cai ratio > 2.5) and systemic ionic hypocalcemia (Cai < 1.0 mmol/l) had higher peak serum lactate, higher infusion rate of calcium and more severe metabolic acidosis. Khadzhynov et al. observed a higher basal lactate level in patients with signs of citrate accumulation^25^. We showed that the correlation between serum citrate and arterial lactate levels was positive and weak (mean during the first 7 days of CVVHDF r = 0.441).

We did not detect a difference between the dialysis dose offered between patients with and without LF. The lifespan of the filters, the number of filters used per patient, the number of replacements due to coagulation and the treatment interruption time were not statistically significant between the groups with and without LF. By limiting citrate infusion in the LF group, higher levels of post-filter ionized Ca were tolerated. This had no impact on the coagulation of the extracorporeal system, probably due to the fact that these individuals presented greater coagulation disorders and lower hemoglobin levels, according to our results.

### Citrate and mortality

The fact that the association between the measured citrate levels and mortality was independent of cirrhosis in our cohort was not observed in other studies. Link et al., using the Cat/Cai ratio as a marker of citrate accumulation, showed that patients with a ratio ≥ 2.4 had a 33-fold increased risk of death compared to patients with values below 2.4^26^. It is not yet clear whether all the observed changes (metabolic acidosis, hypocalcemia, hyperlactatemia) are due to citrate accumulation per se or hypercitratemia is just an indicator of an underlying metabolic derangement in critically ill patients with liver failure, shock, and low tissue perfusion^24,25^.

Mortality in patients with LF is expected to be high, especially with simultaneous kidney failure. Most centers avoid using citrate as an anticoagulant therapy for these severely ill patients due to the possible toxicity of citrate accumulation. However, measures like fixing the citrate infusion rate, keeping a proper dialysis dose, and following the protocol strictly it is possible to use citrate safely even in patients with LF. The correlation between citrate levels with Cat/Cai, INR, bilirubin and lactate is positive, but weak—so the ideal would be to measure citrate directly.

### Study strengths and limitations

Some limitations are typical of observational study designs, and these apply to this work as well: a causal relationship cannot be stablished nor ruled out, and we only observed associations. However, some of the correlation between hypercitratemia and death was independent of other risk factors evaluated, and this should be explored further.

The fact that our study was conducted in one hospital only is both a limitation—as it limits generalizability—and a strength—as we could guarantee methodological homogeneity of data collection and analysis. We invite other researchers to replicate our methods in other centers, and with longer follow-ups: the analysis of laboratory data, especially Ci, was limited to the first week of dialysis, but peaks of changes seem to appear around the third day of therapy, according to the literature^17^.

One could argue against the definition of LF based on INR changes only. However, this variable is used in other clinical conditions such as for calculating the MELD (Model for End-stage Liver Disease) score for organ allocation in liver transplant^27^.

## Conclusions

We showed that hypercitratemia was an independent risk factor for death in individuals undergoing CVVHDF and RCA. Although we observed accumulation of citrate in LV, the increase in levels was limited and without significant clinical significance, even in these patients receiving more transfusion and additional citrate load.

The use of RCA for patients with LF was feasible and safe, once the fixed infusion of citrate was maintained at 2.5 mmol/l of blood and the dialysis dose offered was around 30 ml/kg/h. The correlation between citrate levels and liver function indicators was weak, and this may indicate that measuring citrate levels directly can be more useful for a closer monitoring of patients with liver failure in CVVHDF and RCA.

It is not possible to establish a direct cause and effect relationship between citrate level and mortality. Hypothetically, this association could only indicate a severe metabolic derangement in critically ill patients.

### Supplementary Information

Supplementary Information.

## Data Availability

All data generated or analyzed during this study are included in this article and its supplementary material files. Further enquiries can be directed to the corresponding author.
