# Supplementary material for: Hypercitratemia is a mortality predictor among patients on continuous venovenous hemodiafiltration and regional citrate anticoagulation
Source: Sci Rep. 2023 Nov 17;13:20176. doi: 10.1038/s41598-023-47644-1 (PMC10656486; doi:10.1038/s41598-023-47644-1)
Supplement: Supplementary file 1 — Supplementary Information. [file 41598_2023_47644_MOESM1_ESM.docx]

**Supplementary Table S1.** Serum markers according to liver failure in the third day of dialysis (medians and interquartile ranges)

|  | **Liver failure** | | |  |
| --- | --- | --- | --- | --- |
|  | **Yes (52)** | **No (108)** | **Total (162**)** | **p-value** |
| Creatinine (mg/dL) | 1.3 (0.99-1.64) | 1.19 (0.9-1.55) | 1.23 (0.9-1.6) | 0.190 |
| Urea (mg/dL) | 40 (30-60) | 40 (29-54) | 40 (29-57) | 0.431 |
| Basal sodium (mEq/L) | 141 (138-143) | 140 (138-142) | 140 (138-142) | 0.174 |
| Basal potassium (mEq/L) | 3.7 (3.4-3.8) | 3.7 (3.5-4.0) | 3.7 (3.5-3.9) | 0.020 |
| Chlorine (mEq/L) | 104 (102-106) | 104 (101-106) | 104 (102-106) | 0.206 |
| Anion gap | 13 (10-16) | 11 (9-13) | 11 (9-14) | 0.019 |
| Systemic ionic calcium (mmol/L) | 1.17 (1.12-1.20) | 1.16 (1.13-1.19) | 1.16 (1.13 – 1.19) | 0.241 |
| Post-filter ionized calcium (mmol/L) | 0.45 (0.42-0.49) | 0.34 (0.31-0.36) | 0.35 (0.32-0.43) | <0.001 |
| Total calcium (mg/dL) | 8.9 (8.3-9.4) | 8.8 (8.3-9.2) | 8.8 (8.3-9.2) | 0.384 |
| Phosphor (mg/dL) | 3.7 (3.3-4.3) | 3.6 (3.1-4.2) | 3.6 (3.2-4.2) | 0.288 |
| Magnesium (mg/dL) | 1.7 (1.6-1.8) | 1.7 (1.6-1.8) | 1.7 (1.6-1.8) | 0.583 |
| pH | 7.41 (7.36-7.45) | 7.43 (7.38-7.47) | 7.43 (7.37-7.46) | 0.060 |
| Bicarbonate (mEq/L) | 22.9 (20.8-25.2) | 25.3 (22.7-27) | 25 (22-27) | <0.001 |
| Lactate (mg/dL) | 22 (16-33) | 15 (11-22) | 17 (12-26) | <0.001 |
| AST (U/L) | 671 (107-2823) | 72 (39-181) | 93 (48-443) | <0.001 |
| ALT (U/L) | 799 (129-2491) | 55 (35-171) | 102 (41-529) | <0.001 |
| Total bilirubin (mg/dL) | 8.3 (5.3-13.6) | 2.3 (1.0-4.5) | 4.6 (2-9.6) | <0.001 |
| Direct bilirubin (mg/dL) | 5.5 (3.6-8.0) | 1.6 (0.6-3.3) | 3.1 (1.4-6.5) | <0.001 |
| INR | 2.52 (1.92-4.18) | 1.26 (1.13-1.49) | 1.4 (1.2-2.1) | <0.001 |
| TTPA | 1.76 (1.48-2.23) | 1.25 (1.11-1.47) | 1.43 (1.19-1.71) | <0.001 |
| Hemoglobin (g/dL) | 8.2 (7.5-9.5) | 8.8 (8.1-9.8) | 8.7 (7.8-9.7) | 0.012 |
| Hematocrit (%) | 23.2 (21.3-26.5) | 25.9 (23.2-28.5) | 25.4 (22.7-27.9) | 0.001 |
| Platelets x 10^3^ (n/µL) | 34 (23-61) | 75.5 (32.3-145) | 54 (29-123) | <0.001 |

AST: aspartate aminotransferase; ALT: alanine aminotransferase; INR: international normalized ratio; APPT: activated partial thromboplastin time ratio.

**Total sample reduced due to patients’ deaths.

**Supplementary Table S2.** Serum markers according to liver failure in the seventh day of dialysis (medians and interquartile ranges)

|  | **Liver failure** | | |  | | |
| --- | --- | --- | --- | --- | --- | --- |
|  | **Yes (30)** | **No (51)** | **Total (81**)** | | **p-value** |  |
| Creatinine (mg/dL) | 1.12 (0.93-1.34) | 0.95 (0.78-1.23) | 0.99 (0.8-1.27) | | 0.092 |  |
| Urea (mg/dL) | 46 (35-59) | 43 (28-54) | 44 (30-55) | | 0.205 |  |
| Basal sodium (mEq/L) | 142 (139-143) | 140 (138-143) | 141 (138-143) | | 0.206 |  |
| Basal potassium (mEq/L) | 4.0 (3.6-4.2) | 4.0 (3.8-4.3) | 4(3.8-4.3) | | 0.334 |  |
| Chlorine (mEq/L) | 104 (101-105) | 104 (102-105) | 104 (102-105) | | 1.0 |  |
| Anion gap | 13 (10-15) | 10 (9-13) | 12 (9-14) | | 0.008 |  |
| Systemic ionic calcium (mmol/L) | 1.15 (1.10-1.22) | 1.17 (1.13-1.19) | 1.17 (1.12-1.20) | | 0.730 |  |
| Post-filter ionized calcium (mmol/L) | 0.42 (0.35-0.38) | 0.34 (0.32-0.36) | 0.35 (0.31-0.41) | | <0.001 |  |
| Total calcium (mg/dL) | 9.1 (8.3-9.8) | 8.8 (8.4-9.3) | 8.9 (8.4-9.5) | | 0.377 |  |
| Phosphor (mg/dL) | 3.9 (3.4-4.9) | 3.9 (3.5-4.3) | 3.9 (3.5-4.5) | | 0.536 |  |
| Magnesium (mg/dL) | 1.8 (1.7-1.8) | 1.7 (1.6-1.8) | 1.7 (1.6-1.8) | | 0.027 |  |
| pH | 7.46 (7.37-7.48) | 7.45 (7.41-7.49) | 7.45 (7.41-7.49) | | 0.756 |  |
| Bicarbonate (mEq/L) | 25.1(24.4-26.4) | 26 (24.9-27.5) | 25.6 (24.6-27.2) | | 0.08 |  |
| Lactate (mg/dL) | 18 (14-25) | 12 (9-19) | 14 (11-21) | | 0.012 |  |
| AST (U/L) | 142 (94-231) | 57 (34-64) | 88 (50-199) | | 0.002 |  |
| ALT (U/L) | 369 (95-462) | 60 (33-199) | 104 (48-387) | | <0.001 |  |
| Total bilirubin (mg/dL) | 8.6 (4.2-16.8) | 1.9 (0.8-6.7) | 5.9 (1.8-11.9) | | <0.001 |  |
| Direct bilirubin (mg/dL) | 7 (2.7-11) | 1.1 (0.7-2.0) | 3.1 (1.0-9.5) | | <0.001 |  |
| INR | 1.78 (1.43-2.7) | 1.19 (1.1-1.26) | 1.24 (1.14-1.67) | | <0.001 |  |
| APPT | 1.44 (1.18-1.68) | 1.22 (1.13-1.45) | 1.26 (1.14-1.51) | | 0.044 |  |
| Hemoglobin (g/dL) | 8.7 (8.0-9.5) | 8.7 (7.9-9.8) | 8.7 (7.9-9.6) | | 0.671 |  |
| Hematocrit (%) | 24.5 (22.9-27.5) | 26.1 (23.2-28.4) | 25.8 (23.1-27.9) | | 0.245 |  |
| Platelets x 10^3^ (n/µL) | 34 (19-75) | 75 (35-175) | 65 (23-113) | | <0.001 |  |

AST: aspartate aminotransferase; ALT: alanine aminotransferase; INR: international normalized ratio; APPT: activated partial thromboplastin time ratio.

**Total sample reduced due to patients’ deaths.

**Table S3.** Absolute and relative frequencies of electrolyte and acid-base changes in patients with and without liver failure

| Lab changes | **Liver failure** | | |  |
| --- | --- | --- | --- | --- |
|  | **Yes (417)** | **No (850)** | **Total (1267)** | **p-value** |
| pH ≥ 7.50 | 26 (10.8) | 82 (17.5) | 108 (15.3) | 0.034 |
| pH ≤ 7.20 | 10 (4.2) | 14 (3.2) | 25 (3.5) | 0.099 |
| Bic ≥ 30 | 5 (1.7) | 25 (4.2) | 30 (3.3) | 0.049 |
| Bic ≤ 15 | 43 (14.4) | 27 (4.5) | 70 (7.8) | <0.001 |
| Na ≥ 150 | 2 (0.7) | 9 (1.5) | 11 (1.2) | 0.285 |
| Na ≤ 130 | 13 (4.3) | 15 (2.5) | 28 (3.1) | 0.134 |
| K ≥ 6.0 | 0 (0) | 1 (0.2) | 1 (0.1) | 0.018 |
| K ≤ 3.0 | 5 (1.5) | 18 (2.8) | 23 (2.4) | 0.224 |
| P ≥ 6.0 | 11 (4.5) | 15 (3.2) | 26 (3.7) | 0.094 |
| P ≤ 2.0 | 1 (0.3) | 4 (0.7) | 5 (0.6) | 0.505 |
| Cai ≥ 1.45 | 1 (0.3) | 1 (0.2) | 2 (0.2) | 0.980 |
| Cai ≤ 1.0 | 21 (6.5) | 30 (4.6) | 51 (5.2) | 0.136 |
| Mg ≥ 2.5 | 1 (0.3) | 5 (1) | 6 (0.7) | 0.195 |
| Mg ≤ 1.0 | 1 (0.3) | 2 (0.4) | 3 (0.3) | 0.740 |

pH: arterial serum pH (reference value: 7.25-7.35); Bic: serum bicarbonate in mEq/l (reference value 22-26 mEq/l); Na: serum sodium in mmol/l (reference value: 135-145 mmol/l); K: serum potassium in mEq/l (reference value: 3.5-5.5mEq/l); P: serum phosphorus in mg/dL (reference value: 2.5-4.5mg/dL); Cai: serum ionized calcium in mmol/l (reference value: 1.12-120mmoL/L); Mg: serum magnesium in mg/dl (reference value: 1.7-2.6mg/dl).

**Table S4.** Median and interquartile ranges (Q1-Q3) of the ratio between total and ionized calcium between the first and seventh days of dialysis

| **Total/ionic calcium ratio** | **Liver failure** | | | | |  |  |  | |
| --- | --- | --- | --- | --- | --- | --- | --- | --- | --- |
|  | **Yes** | **N** | **No** | **N** | **Total** | | **N** | | **p-value** |
| First day | 1.92 (1.8-2.02) | 61 | 1.86 (1.76-1.98) | 136 | 1.88 (1.78-2.0) | | 197 | | 0.035 |
| Second day | 1.89 (1.8-2.12) | 55 | 1.86 (1.81-1.99) | 128 | 1.82 (1.81-2.01) | | 183 | | 0.193 |
| Third day | 1.93 (1.83-2.05) | 51 | 1.92 (1.8-2.0) | 102 | 1.92 (1.81-2.01) | | 153 | | 0.309 |
| Fourth day | 1.94 (1.8-2.02) | 43 | 1.88 (1.81-1.97) | 81 | 1.91 (1.81-2.0)­ | | 124 | | 0.125 |
| Fifth day | 1.93 (1.82-2.05) | 36 | 1.8 (1.81-2.02) | 67 | 1.9 (1.81-2.02) | | 103 | | 0.281 |
| Sixth day | 1.93 (1.84-2.06) | 35 | 1.91 (1.83-1.98) | 65 | 1.92 (1.83-2.02) | | 100 | | 0.209 |
| Seventh day | 1.96 (1.86-2.12) | 28 | 1.9 (1.8-1.98) | 47 | 1.93 (1.84-2.02) | | 75 | | 0.066 |

**Table S5.** Correlation between daily serum citrate measurements (mg/dl) and the ratio between total calcium and ionized calcium during each day of dialysis

|  | **Spearman correlation coefficient** | **p-value** | **n** |
| --- | --- | --- | --- |
| First day | 0.337 | <0.001 | 173 |
| Second day | 0.297 | <0.001 | 168 |
| Third day | 0.257 | 0.002 | 146 |
| Fourth day | 0.354 | <0.001 | 122 |
| Fifth day | 0.283 | 0.005 | 97 |
| Sixth day | 0.258 | 0.016 | 87 |
| Seventh day | 0.199 | 0.099 | 70 |

**Table S6.** Correlation between daily serum citrate measurements (mg/dl) and the international normalized ratio (INR) during each day of dialysis

|  | **Spearman correlation coefficient** | **p-value** | **n** |
| --- | --- | --- | --- |
| First day | 0.405 | <0.001 | 176 |
| Second day | 0.366 | <0.001 | 167 |
| Third day | 0.379 | <0.001 | 146 |
| Fourth day | 0.440 | <0.001 | 121 |
| Fifth day | 0.438 | <0.001 | 103 |
| Sixth day | 0.358 | 0.002 | 91 |
| Seventh day | 0.422 | 0.002 | 72 |

**Table S7.** Correlation between daily serum citrate measurements (mg/dl) and serum total bilirubin during each day of dialysis

|  | **Spearman correlation coefficient** | **p-value** | **n** |
| --- | --- | --- | --- |
| First day | 0.357 | <0.001 | 176 |
| Second day | 0.383 | <0.001 | 167 |
| Third day | 0.526 | <0.001 | 146 |
| Fourth day | 0.425 | <0.001 | 121 |
| Fifth day | 0.722 | <0.001 | 103 |
| Sixth day | 0.531 | <0.001 | 91 |
| Seventh day | 0.623 | <0.001 | 72 |

**Table S8.** Correlation between daily serum citrate measurements (mg/dl) and arterial lactate during each day of dialysis

|  | **Spearman correlation coefficient** | **p-value** | **n** |
| --- | --- | --- | --- |
| First day | 0.499 | <0.001 | 176 |
| Second day | 0.381 | < 0.001 | 167 |
| Third day | 0.231 | 0.026 | 146 |
| Fourth day | 0.389 | <0.001 | 121 |
| Fifth day | 0.478 | <0.001 | 103 |
| Sixth day | 0.560 | <0.001 | 91 |
| Seventh day | 0.552 | <0.001 | 72 |

**Table S9.** Correlation between daily serum citrate measurements (mg/dl) aspartate aminotransferase (AST) during each day of dialysis

|  | **Spearman correlation coefficient** | **p-value** | **n** |
| --- | --- | --- | --- |
| First day | 0.240 | 0.075 | 176 |
| Second day | 0.101 | 0.378 | 167 |
| Third day | 0.376 | 0.008 | 146 |
| Fourth day | 0.065 | 0.709 | 121 |
| Fifth day | 0.153 | 0.457 | 103 |
| Sixth day | 0.032 | 0.892 | 91 |
| Seventh day | 0.235 | 0.418 | 72 |

**Table S10.** Demographic and clinical characteristics according to death

|  | **Death** | | |  |
| --- | --- | --- | --- | --- |
|  | **Yes (115)** | **No (85)** | **p-value** | |
| Male sex | 74 (64.9) | 51 (60) | 0.072 | |
| Age | 66 (56-79) | 66 (53-77) | 0.618 | |
| White ethnicity | 103 (89.6) | 75 (88.2) | 0.766 | |
| SAPS-3 ^a^ | 67 (58-80) | 60 (46-67) | <0.001 | |
| SOFA at CVVHDF start | 14 (12-16) | 12 (10-14) | <0.001 | |
| CKD | 10 (8.7) | 7 (8.2) | 0.908 | |
| Hypertension | 37 (32.2) | 30 (35.3) | 0.644 | |
| Diabetes | 30 (26.1) | 21 (24.7) | 0.825 | |
| COPD | 18 (15.7) | 6 (7.1) | 0.064 | |
| Solid malignant tumor | 19 (16.5) | 10 (11.8) | 0.345 | |
| Hematological cancer | 20 (17.4) | 6 (7.1) | 0.032 | |
| HSCT | 8 (7) | 2 (2.4) | 0.140 | |
| Cirrhosis | 31 (27) | 11 (12.9) | 0.016 | |
| Liver transplant | 14 (12.2) | 21 (24.7) | 0.021 | |
| Heart transplant | 2 (1.7) | 1 (1.2) | 0.746 | |
| Kidney transplant | 3 (2.6) | 4 (4.7) | 0.425 | |
| **Reason for admission to the ICU** |  |  |  | |
| Sepsis | 74 (64.3) | 39 (45.9) | 0.009 | |
| Vasoactive drug | 102 (88.7) | 67 (78.8) | 0.057 | |
| Mechanical ventilation | 99 (86.1) | 59 (69.4) | 0.004 | |
| Sedation | 92 (80) | 56 (65.9) | 0.024 | |
| Oliguria | 84 (73) | 47 (55.3) | 0.009 | |
| Liver failure | 72 (62.6) | 19 (22.4) | 0.023 | |

Categorical variables shown as absolute and relative frequencies; continuous variables shown as medians and interquartile ranges.

^a^SAPS-3 calculated at ICU admission.

SAPS: simplified acute physiology score; SOFA: sequential organ failure assessment; CVVHDF: continuous venovenous hemodiafiltration; CKD: chronic kidney disease under dialysis; COPD: chronic obstructive pulmonary disease; HSCT: hematopoietic stem cell transplantation; ICU: intensive care unit.

**Figure legends**

**Supplementary Figure 1.** Generalized estimating equation (GEE) model of the variation of serum pH according to liver failure in the seven first days of dialysis.

**Supplementary Figure 2.** Generalized estimating equation (GEE) model of the variation of serum bicarbonate according to liver failure in the seven first days of dialysis.

**Supplementary Figure 3.** Generalized estimating equation (GEE) model of the variation of serum sodium according to liver failure in the seven first days of dialysis.


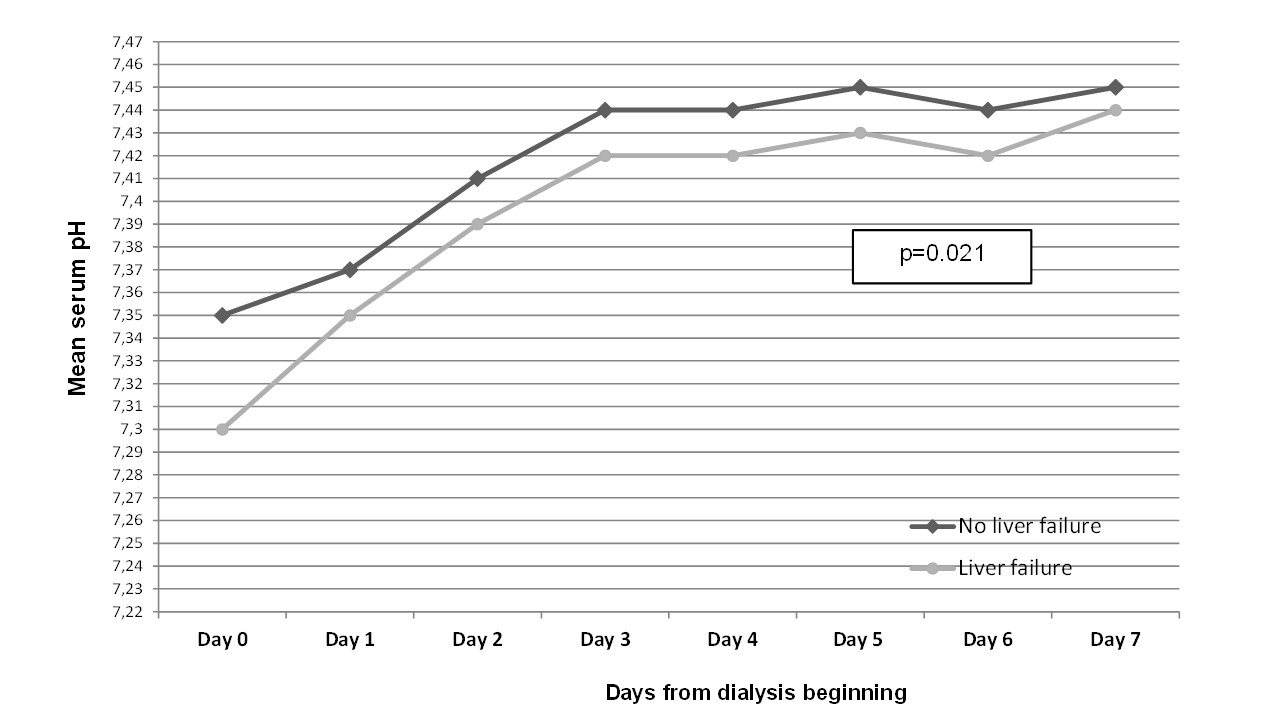


**Supplementary Figure 1.**


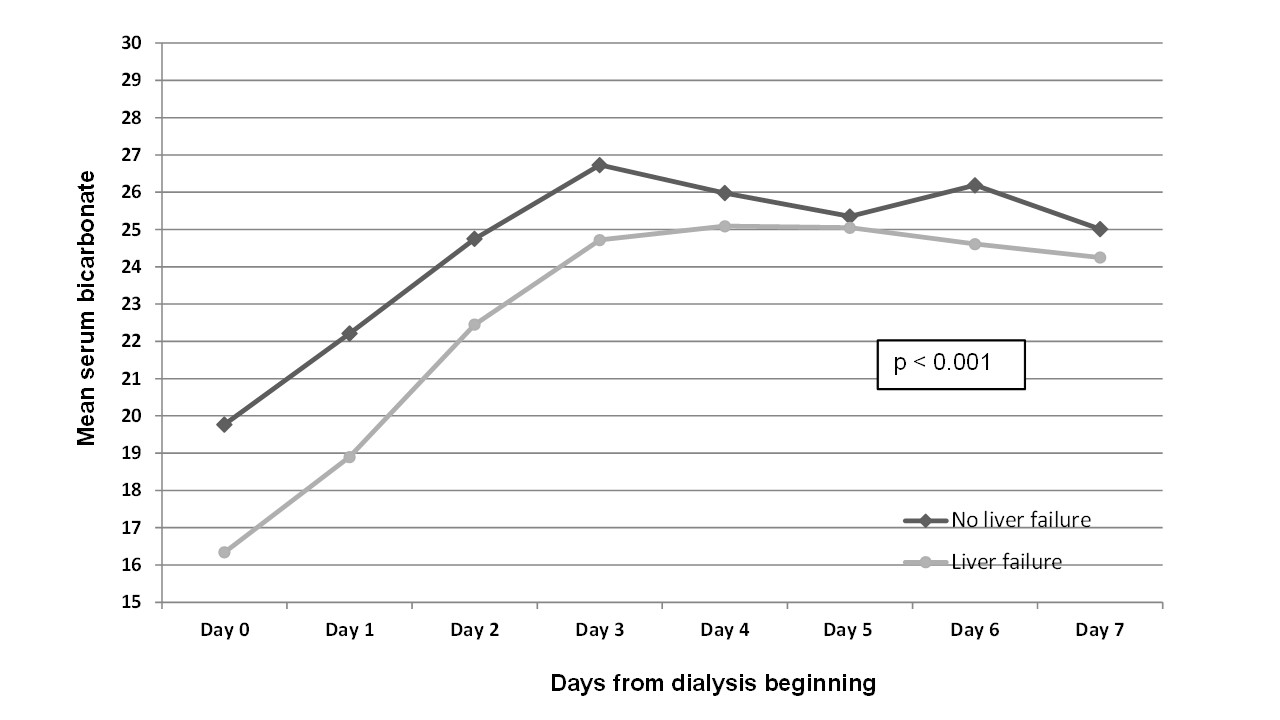


**Supplementary Figure 2.**


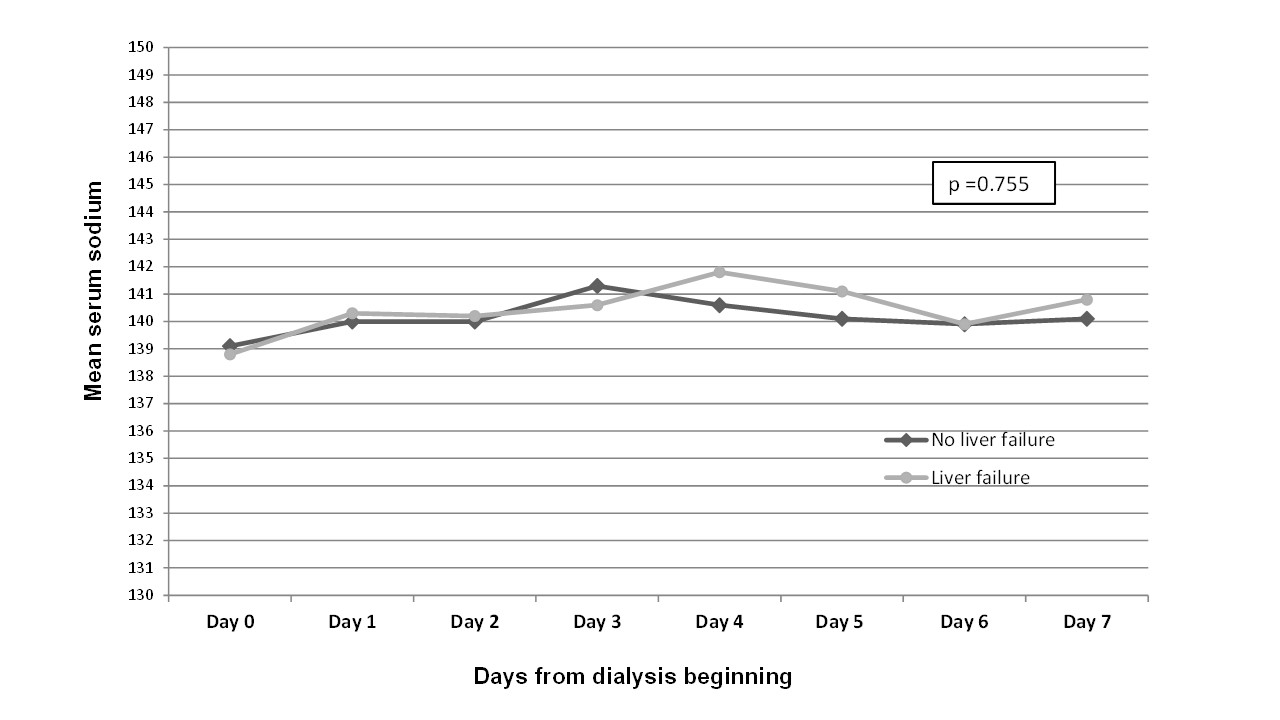


**Supplementary Figure 3.**
